# The Opinion of Polish Coeliac Patients on the Knowledge of Medical Professionals and Support Groups about Coeliac Disease—A Pilot Study

**DOI:** 10.3390/ijerph20053990

**Published:** 2023-02-23

**Authors:** Emilia Majsiak, Magdalena Choina, Bożena Cukrowska

**Affiliations:** 1 Department of Basic Nursing, Chair of Development in Nursing, Faculty of Health Promotion, Medical University, Staszica 4/6, 20-081 Lublin, Poland; 2 Polish-Ukrainian Foundation of Medicine Development, Nałęczowska 14, 20-701 Lublin, Poland; 3 Department of Pathomorphology, The Children’s Memorial Health Institute, Aleja Dzieci Polskich 20, 04-730 Warsaw, Poland

**Keywords:** coeliac disease, gluten-free diet, general practitioners, healthcare professionals (HSPs), primary care professionals (PCPs)

## Abstract

A high quality of knowledge and how it is communicated by healthcare professionals (HCPs) let the patient understand coeliac disease (CD) and result in better adherence to therapeutic recommendations. Therefore, the aim of the current study was to assess the opinion of Polish respondents with CD on the comprehension of CD among Polish HCPs. The analysis was based on 796 responses from patients (the members of the Polish Coeliac Society) with confirmed CD diagnosis (224; 28.1% children and 572; 71.9% adults). The most frequently consulted HCPs regarding CD symptoms in the analysed group were gastroenterologists, and various support groups and associations for CD patients. Furthermore, their comprehension of CD was rated best, as 89.3% (n = 552) of the patients who had contact with support groups and associations classified their knowledge on CD as good. More than a half of the respondents (n = 310, 56.6%) who had contact with general practitioners (GPs) due to their symptoms, rated the doctor’s knowledge on CD as bad. Nurses’ comprehension on CD was classified as bad by 45 (52.3%) respondents who had contact with a nurse. Out of 294 Polish patients with CD who had contact with a dietician, 247 (84.0%) assessed that the dietician communicated their knowledge on CD well. The respondents rated that GPs and nurses communicated their knowledge on CD in the worst manner (60.4% and 58.1%, respectively). Out of 796 respondents, 792 (99.5%) provided information about the number of appointments with GPs due to symptoms that occurred prior to CD diagnosis. The respondents had contact with GPs 13 863 times before obtaining a CD diagnosis due to their symptoms. After the establishment of a CD diagnosis, the number of appointments with GPs decreased to 3850, and the average number of appointments decreased from 17.8 to 5.1. The respondents assessed that the knowledge on CD of HCPs is not satisfactory. The work of support groups and associations on CD, who promote reliable CD diagnosis and treatment methods, should be promoted. The cooperation between various HCPs needs to be encouraged, which may lead to better compliance.

## 1. Introduction

Coeliac disease (CD) is a lifelong systemic autoimmune disorder, provoked by gluten and related prolamins in genetically predisposed individuals [1]. Being one of the most common autoimmune diseases (its prevalence in most populations is estimated to be 1%, and is increasing), CD still poses a challenge to clinicians [2]. It is widely believed that CD is a paediatric disease that manifests only as a malabsorption syndrome. Nevertheless, the clinical manifestation of CD is various, and CD can occur at any age [3]. Although it is one of the most common autoimmune diseases, CD remains undiagnosed for a long time in many patients. It was demonstrated that in Polish patients the mean duration of symptoms prior to CD diagnosis was 7.3 years [4]. Other studies reported that CD diagnostic delay lasted a decade or even longer [2,5].

Although the general awareness of CD has risen, the disease is still underdiagnosed [6]. Early CD diagnosis and introduction of suitable treatment, i.e., a gluten-free diet (GFD), are crucial to prevent patients from serious complications [2,3]. Activities that aim to shorten the diagnostic process are of great importance, as the recognition of CD and elimination of gluten from the diet resulted in significant improvement of quality of life (QoL) among Polish respondents with CD [4]. Thus, it is necessary to determine the causes of the diagnostic delay in CD. One of the possible reasons for the prolonged diagnostic process could be poor knowledge about CD among healthcare professionals (HCPs). Nevertheless, more attention should be paid to primary care professionals’ (PCPs) education, as an appointment with them is often the first step in the CD diagnostic process. Further awareness-raising and educational activities may result in a shorter diagnostic process for CD and improved QoL in CD patients. Increased awareness of CD among general practitioners (GPs) and active case-finding are recognised means to reduce costs spent on CD diagnosis [7].

The available studies, which assess the knowledge of HCPs on CD based on questionnaires, show that the knowledge concerning the diagnostic process, treatment and prevention of CD complications is inappropriate to the healthcare service’s demands [3,8,9]. They also indicate the urgent need for updating the schedule of education of HCPs to improve their abilities [8]. The work of HCPs is addressed to a patient, who can rate both the knowledge and the ability to share the knowledge. The high quality of the knowledge and how it is communicated let the patient understand the disease and result in better adherence to therapeutic recommendations and to a GFD, which prevents the patient from experiencing CD complications. As our research has shown, despite a GFD, CD symptoms persist in more than 80% of Polish patients [10]. One possible reason could be that patients did not follow the therapeutic recommendations, because they did not trust their HCPs, or the way in which recommendations were communicated was not clear enough. Therefore, we wanted to reveal the opinion of CD patients on how they assessed the knowledge about CD among HCPs and support groups and the way it was passed on. No studies assessing the knowledge on CD among Polish HCPs by patients with CD are available.

Our previous research has shown that diagnosing CD is still challenging in Poland [4,10]. Although the time from onset of the symptoms to CD diagnosis is shorter than in the United Kingdom, the findings are unsatisfactory. The diagnostic delay in CD can be explained by the organization of the Polish health care system, the lack of adequate reimbursement of tests to diagnose CD recommended by the guidelines in Poland, and the costs that the patient must incur in order to be diagnosed with CD. However, is this the only thing that can affect the diagnostic delay? Can the assessment of the patients bring benefits to the services provided by HCPs? Can the opinion of patients provide feedback on how they perceive the knowledge and the way it is passed on by HCPs regarding therapeutic and educational recommendations in the use of a GFD?

Various HCPs and support groups for people with CD can be engaged in the diagnosis and then treatment of the disease. Among HCPs, we can distinguish professionals directly involved in diagnosing coeliac disease (such as general practitioners and gastroenterologists), and medical staff not involved in the diagnosis, who may have contact with the CD patient (such as dieticians and nurses). The opinions of patients on HCPs’ knowledge about CD and the way HCPs pass on the information, play a pivotal role in forming a relationship with and gaining confidence in HCPs. Loss of confidence in specific HCPs may lead to refusal of therapeutic recommendations and a restrictive GFD. Furthermore, mistrust in HCPs may encourage belief in celebrities or influencers, in other words self-proclaimed “opinion leaders of healthcare”, who pass unverified, untrue information. If the patients assess that the knowledge of HCPs is unsatisfactory and that HCPs do not pass on their knowledge appropriately, they will search for help from non-specialists. Meanwhile, support groups associate patients with a specific type of disease, e.g., CD, and enable the patients to share experiences or exchange information with other patients.

The aim of the study was to evaluate the opinion of Polish respondents with CD on the assessment of the knowledge on the disease and the way it is passed on by HCPs and support groups. Additionally, in the study we also checked how many appointments patients had to make to be diagnosed with CD. The number could help to estimate and reduce the costs of diagnostic delay incurred by the healthcare system in Poland.

## 2. Materials and Methods

### 2.1. Study Design

A sample of 2500 members of the Polish Coeliac Society received a letter with information on the planned study and a request to complete the attached questionnaire. Of this number, 969 (38.76%) surveyees returned the questionnaire. Out of 969 surveyees, 796 respondents were included to the study group and 172 (17.85%) did not meet the inclusion criteria. These respondents were disqualified by the authors of the study because they introduced a GFD without a proper CD diagnosis. Parents or guardians completed the questionnaire on behalf of children (under the age of 18). The study was conducted with the consent of the Bioethics Committee of the Children’s Memorial Health Institute (No. 48 /KBE/2017).

### 2.2. Questionnaires

The questions underlying the analysis in this study were added to the original questionnaire developed by Gray and Papanicolas, entitled “The Impact of Coeliac Disease on Your Life: A Survey of Your Views”, and was adapted and used with the consent of the authors [5]. The questionnaire included questions about socio-demographic factors, diagnostic and clinical aspects and CD-related costs. In comparison to the original British questionnaire, the Polish one was modified. Particularly, a question concerning the decision to follow a GFD was added, to allow respondents who adopted a GFD on their own to be excluded. Respondents who answered that a GFD had been adopted after a CD diagnosis were then asked about the method of diagnosis: serological test (without specification of the test), duodenal biopsy, genetic examination or any other type of examination, which they were asked to specify. Surveyees were also asked to indicate the specialization of the clinician who had established the CD diagnosis. Respondents were asked about which HCP specialists (GPs, nurses, nutritionists, gastroenterologists) and support groups they had contact with. In the Polish version of the questionnaire, questions regarding patients’ opinions on the knowledge about CD and the way it was passed on by HCPs and support groups were introduced. Respondents were asked to assess the knowledge of HCPs and support groups with a 3-point scale: good, appropriate and bad. They were also asked to assess the manner of passing on the knowledge on CD in terms of good or bad. Another question asked about the number of visits to individual HCPs.

After translation into Polish, all added questions were validated by pre-testing. Then the questionnaire was improved and retested to remove the shortcomings. In order to verify the reproducibility and reliability of the method, the results were interpreted with the characteristics of the tested sample and its size. The detailed description of the original questionnaire was presented in another paper [4,10].

### 2.3. Statistical Analysis

Statistical analysis was performed using Statistica 10 (StatSoft Poland, Kraków, Poland). Statistically significant correlations between qualitative variables were defined with the Spearman test. The correlation between variables was measured with Spearman’s rank correlation coefficient. Continuous variables were summarised using mean values, whereas variability around mean values was reported in terms of standard deviations (SD). The Wilcoxon test was used to determine whether differences in the number of visits before and after CD diagnosis were significant. The precision around mean values was described with 95% confidence intervals (CI). A *p*-value < 0.05 was considered as statistically significant.

## 3. Results

### 3.1. Characteristics of the Study Group

A response rate of 38.76% was achieved with 796 questionnaires returned (Table 1). In the study group, there were 224 (28.14%) children and 572 (71.86%) adults. A majority of respondents were female (n = 642, 80.7%). The average age of patients at CD diagnosis was 24.1 years (range 1 to 75) and the mean age at survey was 29.4 years (range 2 to 80). The mean duration of any symptom prior to CD diagnosis was 7.3 years for the whole study group. There was a statistically significant difference in mean duration of symptoms between adults and children (9.0 and 3.1 years, respectively, *p* < 0.001). In the analysed group, the symptom that on average lasted for the shortest time prior to CD diagnosis (4.7 years) was diarrhoea, whereas the longest-lasting symptom prior to diagnosis was anaemia (9.2 years). The longest reported duration of diagnosed CD was 53 years. A more detailed description of the group can be found in other studies from our centre [4,10].

### 3.2. The Knowledge of Medical Professions on CD in the Opinion of the Respondents

In the analysed group, the most frequently consulted HCPs were gastroenterologists (n = 733, 92.1%) (Table 2). Then, patients indicated that they consulted support groups (n = 618, 77.6%). The knowledge on CD of support groups was good in the opinion of 89.3% of the Polish surveyees, and 55% of gastroenterologist presented a good level of knowledge on CD. Only one surveyee did not indicate that the knowledge on CD of various support groups for CD patients was good or appropriate. Out of 796 Polish patients with confirmed CD, less than one fourth (n = 190) were referred to a dietician. Despite the low rate of referrals to dieticians, more than one third of Polish respondents had contact with a dietician (n = 294, 36.9%) and in this group more than a half classified dieticians’ knowledge on CD as good (n = 172, 58.5%). However, only 19 surveyees (19/294; 6.46% of the respondents who chose the answer “I had contact”; 19/796; 2.39% of all respondents) saw a dietician regularly, and in this group 16 respondents rated the dieticians’ comprehension of CD as good. More than a half of the respondents (n = 310, 56.6%) who had contact with GPs due to their symptoms, rated the doctor’s knowledge on CD as bad. There was a statistically significant difference in the assessment of knowledge and the way it was communicated by a gastroenterologist and a paediatric gastroenterologist (*p* < 0.001). Parents (or guardians) of children most often assessed the knowledge of paediatric gastroenterologists as sufficient (66.2%) or good (25.0%). Less frequently, the knowledge of a gastroenterologist who sees adults was assessed as sufficient (47.9%) or good (34.5%). There was no statistically significant difference in the assessment of knowledge on CD of GPs and paediatricians (*p* = 0.574, Table 3). Nurses’ understanding of CD was classified as bad by 45 (52.3%) respondents who had contact with a nurse.

Not only the understanding, but also the communication of knowledge on CD was assessed by the respondents. Out of 294 Polish patients with CD who had contact with a dietician, 247 (84.0%) assessed that the dietician passed on their knowledge on CD well. Almost all (n = 610, 98.7%) surveyees stated that they received the most satisfying information on CD from various CD support groups and associations. The way in which paediatric gastroenterologists shared their knowledge on CD with the patients was more often rated as good (78.9%) in comparison with gastroenterologists seeing adults (68.6%) (a statistically significant difference (*p* = 0.002). No statistically significant difference in the way the knowledge on CD was communicated by GPs and paediatricians was revealed (*p* = 0.274, Table 3). The respondents rated that GPs and nurses passed their knowledge on CD in the worst manner (60.4% and 58.1%, respectively).

Out of 796 respondents, 792 (99.5%) provided information about the number of appointments with GPs due to symptoms that occurred prior to CD diagnosis. The mean number of appointments with GPs was 17.8 (Table 4). Analysis with the Spearman’s rank correlation coefficient revealed a statistically significant relationship between the average duration of all symptoms and the number of visits (*p* < 0.001) (Table 5). That is to say, the longer the symptoms lasted, the greater the number of visits (Table 4). Nonetheless, no statistically significant difference between children and adults regarding the number of appointments with GPs due to symptoms prior to CD diagnosis was shown.

The respondents had contact with GPs 13,863 times before obtaining a CD diagnosis due to their symptoms. Out of this number, 9469 appointments were made by adults and 4394 were made by children. After the establishment of a CD diagnosis, the number of appointments with GPs decreased to 3850, and the average number of appointments decreased from 17.8 to 5.1. A total of 567 respondents reported that after their CD diagnosis the number of appointments with GPs decreased, 94 reported that the number increased and 85 respondents did not report any difference. Regardless of the age of the respondent (the whole study group, both children and adults), we noted a statistically significant decrease in the number of appointments with HCPs after the establishment of a CD diagnosis and introduction of a GFD (*p* < 0.001) (Table 6 and Table 7).

## 4. Discussion

The poor knowledge on CD among HCPs may result in under diagnosis of the disease [8,9]. In one of our previous papers, we revealed that the mean duration of CD symptoms prior to diagnosis in Poland was 7.3 years [10]. One of the causes of diagnostic delay may be insufficient knowledge and the way it is communicated by HCPs to patients in the process of diagnosing and treating this disease [3,9]. Therefore, this astonishing diagnostic delay led us to assess how people with recognised CD in Poland evaluate HCPs knowledge on CD.

It is worth noting that the almost one fifth of the Polish respondents (17.85%) introduced a GFD without a doctor’s recommendation [4]. These individuals were excluded from the analysis. Nevertheless, the widespread trend of GFDs may result in more people who start eating gluten-free products without proper diagnosis. In such patients, the CD diagnostic process is particularly difficult as it requires a gluten challenge at an appropriate dose for approximately 6–8 weeks [1].

The respondents were asked to answer questions about whether they had contact with GPs, gastroenterologists, dieticians and nurses, as well as support groups or associations for CD patients. Polish CD patients most frequently had contact with a gastroenterologist due to CD symptoms (more than 90%). It is also worth highlighting that the mean number of appointments with GPs due to CD symptoms prior to diagnosis was approximately 18. Although less than one fourth of Polish CD patients were referred to a dietician, almost 90% of those who consulted a dietician rated dieticians’ knowledge on CD as good or appropriate. Gastroenterologists were regarded as well informed on CD, because more than 80% of the respondents answered that their comprehension of the disease was not bad. More than half of the Polish CD patients rated that GPs and nurses did not have appropriate knowledge on CD. The last aspect assessed by members of the study group was the manner in which HCPs passed on their knowledge on CD. Again, the best rated in this aspect were various support groups. More than 80% of Polish CD patients answered that dieticians shared their knowledge on CD adequately. Almost three quarters of the respondents were satisfied with the manner in which gastroenterologists explained various CD aspects. Meanwhile, 60% of the respondents answered that GPs and nurses did not pass on their knowledge on CD well.

In our analysis, almost 85% of respondents did not assess GPs’ knowledge on CD as good. One factor that could lead to this poor result is unawareness of the pervasiveness of CD and lack of knowledge about the proper approach to diagnosing the disease among GPs. Barzegar et al. also stated that almost 90% of doctors needed training in CD diagnosis and treatment. Barzegar et al. implied the need for a training package developed in accordance with the principles of instructional design to reduce the performance gap in specialists [8]. Authors of a study conducted in the USA revealed that PCPs were not aware of the pervasiveness of CD and lacked knowledge about the proper approach to diagnosing the disease. The study showed that the longer the doctor was in clinical practice (more than 10 years), the more likely they were to screen young men with iron deficiency anaemia for CD [11]. The influence of the age of the doctors and their time in clinical practice on the assessment of their knowledge of CD and the way it is passed on, seems to be an intriguing issue. Although in our study we did not assess the time in clinical practice, we revealed that among all doctors, paediatric gastrologists were rated the highest. This finding may be important, taking into account the significant difference in the diagnostic delay of CD between children and adults in Poland, as shown in the above-mentioned research [4,10].

Serology is the first step, before biopsy, in the CD diagnostic process in children and adults. The concentration of specific antibodies for CD decreases with strict adherence to the GFD [12]. After the exclusion of gluten, the intestinal villi are also restored. That is why, before diagnosing CD, serological test and biopsy should be performed on a diet with gluten. Furthermore, only 20% of clinicians would not introduce a gluten-free diet in a patient with positive serologic testing for CD prior to endoscopy [11]. Out of 2440 members of the Coeliac Disease Foundation in the USA, only 11% of surveyees were diagnosed by PCP or received a referral to a gastroenterologist due to suspicion of CD [13]. In another study it was shown that HCPs other than gastroenterologists were less likely to follow guidelines on CD management [14]. Not only do GPs diagnose CD, but they also play a significant role in post-diagnosis care. However, as it was shown in the study by Pritchard et al., more than 75% of patients with confirmed CD did not have a primary care follow-up appointment [15].

In comparison to Polish GPs, gastroenterologists were assessed better by Polish respondents with CD. More than a half of the surveyees rated that their knowledge on CD was good. This subjective opinion of Polish patients is concordant with the results of studies that verified the knowledge of various specialists, including gastroenterologists. In the Iranian study presented by Barzegar et al., among various physicians, gastroenterologists had the best knowledge on CD. Interestingly, Iranian gastroenterologists scored best regarding CD diagnosis (almost 50% received a good score). The authors of this study believe that it was because of their experience and ability to diagnose patients with very mild or typical presentations, which may remain unrecognised by PCPs [8]. However, other authors were alarmed that gastroenterologists’ knowledge on the CD diagnostic process is poor. Although in a study published by Riznik et al., the overall score achieved by gastroenterologists (both paediatric and internal medicine) was higher in comparison with the score achieved by PCPs; only slightly more than one third of internal medicine gastroenterologists answered questions on CD diagnostic procedure appropriately [9]. Authors of this study indicated that paediatric gastroenterologists performed better in the questionnaire than other doctors, which might have been caused by the publication of the ESPGHAN Guidelines [9]. Even though, in a study conducted by Sahin et al. in Turkey, paediatric gastroenterologists also scored highest in a questionnaire on CD, the authors of the study noticed that only about 50% of correct answers were given in the section on diagnostic procedure, and suggested that the physicians did not follow the guidelines entirely [16]. The need for further education of gastroenterologists on CD was clearly shown by authors of a multicentre study in the United Kingdom (UK), who came to the worrisome conclusion that gastroenterologists did not follow guidelines regarding the performance of endoscopy [17]. It is also worth mentioning that more than one third of gastroenterologists in the UK thought that no doctor was required for the management of CD [17].

As the dietary restrictions after CD diagnosis could be shocking, many CD patients need help to navigate through the GFD. An early and effective appointment with a knowledgeable and skilled dietitian, who will increase the patient’s education on the disease, may be essential to improve adherence and establish a healthy, well-balanced diet [18]. In contrast to this ideal picture, we revealed that less than one in four Polish patients were referred to a dietician after CD diagnosis. However, almost 40% of Polish CD patients had contact with dieticians to consult on the GFD, and the vast majority of these respondents rated dieticians’ comprehension on CD as good or appropriate. A survey conducted by Dembiński et al. in 2021 proved that dieticians had the best knowledge on the GFD. The disproportion between the score obtained by doctors (their speciality was not determined) and dieticians, as well as the students of these faculties, is also worth highlighting. Only one third of doctors and three quarters of dieticians gave more than 60% of the correct answers in the survey. The proportion of the students of medicine and the students of dietetics, who had acceptable level of knowledge was similar (26%, and 55%, respectively). These findings show an obvious need for a change in medical education at universities. The better the perception of CD among students, who will become HCPs, the less doubt there will be about adequate diagnosis and management of the disease. The lack of confidence in GFD counselling among Polish HCPs was also demonstrated by Dembiński et al., who indicated that only 10% of the participants believed that their knowledge on GFD was appropriate [19].

The lowest number of Polish CD patients had contact with nurses (slightly more than 10%), and more than a half of them rated nurses’ knowledge on CD as bad. Although nurses do not play a pivotal role in CD diagnosis and treatment, they should be aware of the disease, especially in the light of the fact that CD can be diagnosed at any age, and a nurse is often the first HCP to consult elderly patients [4]. Of note is the fact that less than one fifth of Polish respondents with CD rated nurses’ and GPs’ knowledge on CD as good, and more than half of them rated the knowledge on CD of these HCPs as bad. The number of nurses and doctors who received acceptable scores in the survey performed by Dembiński et al. was also comparable (34% and 33%, respectively) [19]. We found no data on nurses’ and dieticians’ comprehension of CD from other countries, and we would like to encourage other authors to perform further studies on this issue.

These findings are alarming and suggest medical inertia towards CD among primary and secondary care clinicians. As CD patients receive no help from doctors, they search for other sources of knowledge on CD, such as various support groups for CD patients. Support groups allow the patients to discuss the diagnostics, the treatment, the rehabilitation and the drug programmes. They encourage the patients to talk about their treat-ment or help each other in a way that is convenient for them. The surveyees most frequent-ly indicated that they belonged to the Polish Coeliac Society or groups and forums on the internet. The Polish Coeliac Society is the largest public organization that helps people on a GFD in Poland and belongs to the Association of European Coeliac Societies (AOECS). The organization is highly active in improving the quality of life of people on a GFD. There are associations for CD patients which promote recognised and reliable methods of CD diagnosis and treatment, e. g., the Polish Coeliac Society. Employees and volunteers who work in such associations are usually well educated about CD, which was proved by the opinion of Polish patients. Their effort made for CD patients is important and should be supported, which was proved by Riznik et al. In their study, members of CD support groups in countries of Central Europe achieved better scores in a questionnaire on CD than non-members [9]. On the other hand, some associations and support groups spread misinformation on CD, proposing alternative, unrecognised solutions. Their work can be particularly harmful for people suffering from serious diseases, including CD.

The findings of our study are alarming, especially when we consider how Polish CD patients rated the knowledge on CD of PCPs and nurses. Members of these two HCP groups almost always have the first contact with symptomatic patients, and their approach and perception determine further diagnostic steps. Unfortunately, the unawareness among HCPs of the fact that anaemia can be the only symptom of CD in adults (anaemia was also the longest lasting symptom of CD after the diagnosis in Polish adults—11.6 years on average) [10], as well as inappropriate knowledge on CD, may lead to delays in CD diagnosis. The time from the onset of CD symptoms to the establishment of a CD diagnosis is too long (research conducted in Poland revealed it was 7.3 years on average) [4]. These findings cannot be ignored, as persistent CD symptoms reduce the patients’ quality of life (QoL) [4]. We revealed that the establishment of a CD diagnosis and the introduction of the GFD significantly improved the QoL of Polish patients with CD [4].

It has been widely agreed that poor knowledge on CD presented by HCPs contributes to delayed CD diagnosis [8,13,16,20]. On the contrary, Riznik et al. proposed that relatively short diagnostic delays in children with CD in Central Europe could correspond with the appropriate knowledge of paediatric gastroenterologists [9]. These studies prove that the improvement in the awareness of HCPs, resulting in earlier diagnosis of CD, could help to reduce the costs of medical care [8]. Thus, investing in knowledge on CD can be beneficial also from the economical point of view. The comparison of the number of appointments with GPs before CD diagnosis and the number of visits after CD diagnosis revealed a statistically significant reduction. If the cost of these visits and the burden on the state budget were added, it is highly probable that not only the entire primary health care system would benefit from shortening the time of diagnosis, but also the entire society.

Our findings clearly show that an immediate action must be taken so as to improve HCPs’ knowledge on CD. Although the curriculum at medical faculties is demanding, more information on CD could be introduced, so that future HCPs are more aware of CD diagnosis and treatment. Furthermore, after completing their educational period, HCPs lack comprehension and self-confidence in CD management. Thus, the educational needs of HCPs should be assessed and methods of training that comply with these needs should be introduced [8]. One of the means that could help HCPs update their knowledge on CD are systematically published guidelines that can be introduced in real-life practice. As it was suggested by Riznik et al., available ESPGHAN guidelines on CD might have enhanced paediatric gastroenterologists’ awareness on CD, and that was why they scored higher than other physicians [9]. The organisation and introduction of learning activities should be particularly efficient in case of PCPs, who represent the first contact point for patients with symptoms suggestive of CD [9,16]. The rising awareness of CD in this group of HCPs could significantly shorten the diagnostic process [13], especially because in many countries, including Poland, patients wait for a long time to make an appointment with a gastroenterologist. The benefits of investing in knowledge are obvious, and include not only a reduction in CD diagnostic delay, but also higher patient satisfaction and a reduction in health care costs [9]. Cooperation between various HCPs should also be encouraged, e. g., physicians referring CD patients to dieticians, as it may increase patients’ compliance and lead to further improvement in QoL and savings. What may bring additional benefits for CD patients are support groups and associations for CD patients, provided that their work is based on recognised knowledge. The effort of volunteers from such organisations is extremely valuable and needs to be promoted, because it may help the patient to navigate through a new reality, that is to say, living with CD.

In conclusion, we would like to point out that in the analysis of opinions regarding both knowledge and the way it was delivered by HCPs and support groups, GPs and nurses performed the worst. Among doctors, paediatricians and paediatric gastrologists were rated better than their colleagues dealing with adult patients. In addition, the number of appointments with GPs prior to CD diagnosis indicates the need to take immediate action to reduce the number of patient appointments with GPs prior to CD diagnosis. This analysis showed that in the opinion of Polish patients, support groups, such as the Polish Coealic Society, are valuable and need support, because they provide patients with a common space to solve problems related to CD.

## 5. Limitations of the Study

We are cognizant of the limitations of the study. The HCPs’ knowledge on CD was assessed by the patients and it was not measured directly, for example by a questionnaire completed by HCPs. Thus, the results we obtained are not fully objective. The construction of the study did not allow us to distinguish various HCPs groups depending on the time in clinical practice. Furthermore, we could not determine the gender, age, number of specialisations and other information describing HCPs, which would be useful to prepare a more detailed analysis.

Another limitation of the study is the fact that the study group consisted only of members of the Polish Coeliac Society. Consequently, the opinions of CD patients who were not members of the association were not presented.

## 6. Conclusions

To conclude, the respondents assessed that the knowledge on CD of HCPs is not satisfactory. Awareness-raising and educational activities may result in a shorter diagnostic process for CD and improved QoL in CD patients, as well as a reduction in the costs of CD diagnosis. Further actions that aim to raise awareness of CD among HCPs, especially PCPs, are necessary. That is why educational activities should be suited to the needs of HCPs, so that they update their knowledge on CD and diagnose the disease earlier. The work of support groups and associations on CD, who promote reliable CD diagnosis and treatment methods, should be promoted. The cooperation between various HCPs needs to be encouraged, which may lead to better compliance.

## Figures and Tables

**Table 1:** Characteristics of the study group.

Variables		All Patients	Adults			Children		
No. of included questionnaires		796	at the time of the study		at the time of the diagnosis of CD ^1^	at the time of the study		at the time of the diagnosis of CD
			572; (71.86%)		502; (63.07%)	224; (28.14%)		294; (36.93%)
The mean duration of any symptom prior to CD diagnosis (in years)		7.3	9.0			3.1		
			*p* < 0.001					
Sex—No. (%)	Female	642 (80.7%)						
	Male	154 (19.3%)						
Average age at survey in years—Mean (SD ^1^)		29.4 (16.0)		37.2 (11.7)			9.7 (3.8)	
Average age at diagnosis in years—Mean (SD ^1^)		24.1 (15.9)		34.3 (10.6)			6.8 (4.2)	

^1^ SD = standard deviation.

**Table 2:** The opinion of 796 Polish patients with confirmed CD on the knowledge on CD of medical professions (general practitioners, gastroenterologists, nurses, dieticians and support groups and associations for CD patients).

	I Did Not Have Contact (%)	I Had Contact (%)	No Answer (%)	Knowledge on CD ^1^			The Manner of Passing on the Knowledge on CD	
				Good (%)	Appropriate (%)	Bad (%)	Good (%)	Bad (%)
General Practitioner	247 (31.0)	548 (68.8)	1 (0.1)	91 (16.6)	147 (26.8)	310 (56.6)	217 (39.6)	331 (60.4)
Gastroenterologist	63 (7.9)	733 (92.1)	1 (0.1)	403 (55.0)	226 (30.8)	104 (14.2)	532 (72.6)	201 (27.4)
Nurse	709 (89.1)	86 (10.8)	1 (0.1)	15 (17.4)	26 (30.2)	45 (52.3)	36 (41.8)	50 (58.1)
Dietician	501 (62.9)	294 (36.9)	1 (0.1)	172 (58.5)	90 (30.6)	32 (10.9)	247 (84.0)	47 (16.0)
Support groups and associations for CD patients	177 (22.2)	618 (77.6)	1 (0.1)	552 (89.3)	65 (10.5)	1 (0.1)	610 (98.7)	8 (1.3)

^1^ CD = coeliac disease.

**Table 3:** The opinion of 796 Polish patients with confirmed CD on the knowledge on CD of medical professions distinguishing paediatricians, general practitioners, paediatric gastroenterologists and gastroenterologists.

	I Didn’t Have Contact (%)	I Had Contact (%)	No Answer (%)	Knowledge on CD ^1^			Chi Square Test		The Manner of Passing on the Knowledge on CD		Chi Square Test	
				Good (%)	Appropriate (%)	Bad (%)	χ^2^	*p*	Good (%)	Bad (%)	χ^2^	*p*
Paediatricians	102 (34.7)	192 (65.3)	1 (0.0)	56 (29.2)	33 (17.2)	103 (53.6)	1.11	0.574	82 (42.7)	110 (57.3)	1.20	0.274
General Practitioners	145 (28.9)	356 (70.9)	1 (0.2)	91 (25.6)	58 (16.3)	207 (58.1)			135 (37.9)	221 (62.1)		
Paediatric gastroenterologists	10 (3.4)	284 (96.6)	0 (0.0)	71 (25.0)	188 (66.2)	25 (8.8)	25.20	<0.001	224 (78.9)	60 (21.1)	9.23	0.002
Gastroenterologist	52 (10.4)	449 (89.4)	1 (0.2)	155 (34.5)	215 (47.9)	79 (17.6)			308 (68.6)	141 (31.4)		

^1^ CD = coeliac disease.

**Table 4:** The average number of appointments with GPs ^1^ about the symptoms prior to CD diagnosis, by duration of symptoms.

Duration of Symptoms before the Diagnosis in Years	All Patients (n = 796)		Children (n = 294 ^2^)		Adults (n = 502 ^2^)	
	Number of Patients	Mean of Appointments	Number of Children	Mean of Appointments	Number of Adults	Mean of Appointments
<1	94	7.6	63	9.0	31	4.7
1–5	367	13.5	189	15.9	178	11.0
5–10	164	20.2	31	19.8	133	20.3
10–20	124	28.0	9	20.7	115	28.5
>20	43	36.5	0	-	43	36.5
In total	792 ^3^	17.8	293 ^3^	15.3	500 ^3^	19.2

^1^ GPs—General Practitioners CD—coeliac disease. ^2^ The number of children and adults at the age when the diagnosis of CD was made. ^3^ Some respondents did not provide data on the number of appointments with GPs or did not provide data on the duration of symptoms prior to diagnosis. Results are presented as the total number of appointments and arithmetic means.

**Table 5:** Spearman rank correlation coefficient values. The relationship between duration of symptoms before the CD diagnosis in years and the number of appointments with GPs ^1^ prior to the CD diagnosis.

		The Number of Appointments with GPs ^1^ Prior to CD Diagnosis	
Spearman rank correlation	Duration of symptoms before the diagnosis in years	Correlation factor	0.30
		*p* value	<0.001
	Duration of symptoms before the diagnosis in years/children	Correlation factor	0.24
		*p* value	<0.0001
	Duration of symptoms before the diagnosis in years/adults	Correlation factor	0.33
		*p* value	<0.001

^1^ GPs—General Practitioners.

**Table 6:** The number of appointments with GPs ^1^ prior to CD ^2^ diagnosis of the symptoms.

	No. of Respondents Who Answered the Question on No. of Appointments with GPs	Appointments Prior to CD Diagnosis				No. of Respondents Who Answered the Question on No. of Appointments with GPs	Appointments after CD Diagnosis			
		No. of Appointments with GPs	Mean Number of Appointments with GPs	95.0%—The Lower Limit of Confidence Interval for Mean Value	95.0%—The Upper Limit of Confidence Interval for Mean Value		No. of Appointments with GPs	Mean Number of Appointments with GPs	95.0%—The Lower Limit of Confidence Interval for Mean Value	95.0%—The Upper Limit Of Confidence interval for Mean Value
Children	287	4394	15.3	13.0	17.6	272	1384	5.1	4.0	6.2
Adults	492	9469	19.2	17.0	21.5	485	3850	5.1	4.1	6.1
All patients	779 ^3^	13,863	17.8	16.1	19.5	757 ^3^	3850	5.1	4.3	5.8

^1^ GPs—general practitioners, ^2^ CD—coeliac disease. ^3^ The number of patients is different from 796 because not all patients answered the questions on the number of appointments with GPs before and after the establishment of CD diagnosis.

**Table 7:** The difference in number of appointments with GPs ^1^ about CD symptoms before and after CD diagnosis.

	The Number of Appointments with GPs after CD Diagnosis				Wilcoxon’s Test	
	Decreased	Increased	No Change	In Total	Z	*p*
Children	197	38	33	268	−9.94	<0.001
Adults	370	56	52	478	−14.79	<0.001
All patients	567 ^2^	94	85	746	−17.83	<0.001

^1^ GPs—general practitioners, CD—coeliac disease. ^2^ The number of patients is different from 796 because not all patients answered the questions on the number of appointments with GPs before and after the establishment of CD diagnosis.

## Data Availability

The datasets generated and/or analysed during the current study are not publicly available because they are also being used for further ongoing analyses, but are available from the corresponding author on reasonable request.
